# Novedades de la norma ISO 15189:2023

**DOI:** 10.1515/almed-2023-0129

**Published:** 2023-10-27

**Authors:** Isabel de la Villa Porras

**Affiliations:** Jefa del departamento de Sanidad de ENAC, Laboratorios Clínicos, ENAC, Madrid, España

**Keywords:** norma ISO 15189, calidad, laboratorios clínicos

En diciembre de 2022 se publicó una nueva versión de la norma ISO 15189 “Laboratorios clínicos: Requisitos para la calidad y la competencia” [1]. Tras una revisión menor en 2007, se publicó una primera revisión en 2012 con mayor claridad en su contenido [2]. La revisión de 2022 modifica su estructura y engloba los requisitos de pruebas junto al paciente (POCT). Han pasado 20 años desde que se publicó la primera versión habiéndose convertido en este tiempo en la norma de referencia y en la herramienta mayoritariamente utilizada para aportar confianza, a través de la acreditación, en el correcto desempeño de los laboratorios clínicos a nivel mundial.

La elaboración y revisión de la norma ISO 15189 se ha llevado a cabo con la participación de profesionales de todo el mundo, representado a todas las especialidades, mediante la estructura internacional de normalización, ISO (*International Standard Organization*). Esta estructura se apoya en la participación de los organismos de normalización de cada país (en España Asociación Española de Normalización, UNE, www.une.org.) que en el caso de la norma ISO 15189 se desarrolla a través del comité de UNE, Comité Técnico Nacional CTN 129, del cual son vocales las diferentes sociedades científicas, entre las que se encuentra la Sociedad Española de Medicina de Laboratorio (SEQC-ML). Esta estructura de normalización, basada en el consenso de las partes interesadas, es lo que otorga a las normas así elaboradas su valor añadido y su reconocimiento y validez internacional.

En lo que respecta a los cambios incorporados a la nueva versión hay que resaltar en primer lugar que no implican modificaciones fundamentales en sus requisitos, lo cual pone de manifiesto la madurez y el amplio consenso que existe sobre su contenido e implica que la mayoría de los sistemas implantados en los laboratorios clínicos bajo está norma siguen siendo válidos por lo que los laboratorios acreditados no deben acometer modificaciones de calado para adaptarse a ella.

Los principales cambios consisten en su nueva estructura, alineada con la norma ISO/IEC 17025:2017 [3], y en la incorporación de los requisitos para las pruebas realizadas junto al paciente (POCT), que ahora son parte integral de la norma, lo que implica que ISO 22870:2016 [4] será retirada.

A lo largo de toda la norma se pone mayor énfasis en aspectos que ya estaban contemplados en la versión de 2012, como son la seguridad del paciente, el enfoque basado en riesgos y el fomento de la mejora continua.

Un aspecto destacable en esta versión es la formulación del texto de forma menos prescriptiva lo que permite una mayor flexibilidad para cumplir y evidenciar los requisitos y adaptarlo a cada tipo de prueba o disciplina del laboratorio clínico.

Asimismo, y debido al creciente desarrollo de normas ISO en el sector sanitario, en esta norma se han incluido referencias a varias normas ISO específicas de laboratorios clínicos publicadas recientemente y que, no constituyendo requisitos, la complementan y pueden ser de utilidad para los laboratorios (ej.: ISO 22367 – *Aplicación de la gestión de riesgos a los laboratorios clínicos*, ISO 20658:2023 – *Requisitos para la toma y transporte de muestras para análisis en el laboratorio clínico*).

La publicación de la nueva norma tiene un impacto en los laboratorios clínicos acreditados y en aquellos que deseen solicitar la acreditación ya que deberán adecuar sus sistemas a lo establecido en la nueva norma. Para ello, ENAC ha establecido un período de transición (conforme a lo dispuesto por el *International Laboratory Accreditation Cooperation*, ILAC) con una fecha límite del 6 de diciembre de 2025 en la que todas las acreditaciones de los laboratorios clínicos que estén vigentes deberán hacer referencia a la nueva versión de la norma.

Ello significa que antes de dicha fecha los laboratorios deberán haber demostrado el cumplimiento con los requisitos de la norma ISO 15189:2022. Por otra parte, dado que la norma ISO 15189:2022 incorpora los requisitos de la norma ISO 22870:2016, en el momento en que los laboratorios acreditados para análisis junto al paciente demuestren cumplimiento con la nueva norma se actualizará su alcance de acreditación eliminando la referencia a la norma ISO 22870:2016.

## La acreditación de los laboratorios clínicos frente a la norma ISO 15189

La evaluación del cumplimiento de esta norma se lleva a cabo mediante una herramienta establecida a escala internacional, la acreditación^3^, mecanismo independiente y riguroso que tiene como objetivo generar confianza y credibilidad sobre la correcta ejecución de un determinado tipo de actividades.

El proceso de evaluación corre a cargo del organismo nacional de cada país, en España ENAC (Entidad Nacional de Acreditación, www.enac.es). Para que este proceso sea totalmente efectivo, ENAC está integrado en la infraestructura global de la acreditación (a nivel europeo, EA y a nivel internacional, ILAC) a la que pertenece desde su fundación hace ya más de 30 años.

A través de esta estructura se establecen criterios internacionales y mecanismos de homogeneización que todos los organismos de acreditación deben seguir. Además, se han establecido acuerdos internacionales basados en el reconocimiento mutuo de los informes emitidos por las entidades acreditadas facilitando la consecución del objetivo final*: “acreditado una vez, aceptado en todas partes”.* Así, un informe de laboratorio clínico emitido por una entidad acreditada por ENAC será reconocido como igualmente válido y fiable por cualquiera de los más de 100 países con los que ENAC tiene firmados acuerdos de reconocimiento internacional.

Los acuerdos de reconocimiento mutuos de los que ENAC es firmante se mantienen a través de un sistema de *“peer evaluations”*, sistema por el cual ENAC es periódicamente evaluado por grupos auditores provenientes de otros organismos de acreditación europeos o internacionales.

Para asegurar homogeneidad en la evaluación de la norma ISO 15189, ENAC participa en los grupos de trabajo de sanidad establecidos a nivel europeo e internacional (EA e ILAC) junto con representantes del resto de organismos de acreditación, de sociedades científicas europeas y de la industria del diagnóstico *in vitro*.

ENAC, para desarrollar los procesos de acreditación, colabora con las sociedades científicas de cada especialidad a través de Acuerdos de Colaboración que, en el caso de SEQC, se firmó en el año 2005. En virtud de estos acuerdos, la sociedad científica proporciona a ENAC apoyo en aspectos técnicos específicos, en la revisión de documentos de acreditación y en la aportación de candidatos a auditores técnicos de la especialidad.

## References

[1] UNE-EN ISO 15189:2022 (2022). Laboratorios clínicos. Requisitos para la calidad y la competencia (ISO 15189:2022).

[2] UNE-EN ISO 15189:2013 (2013). Laboratorios clínicos. Requisitos para la calidad y la competencia (ISO 15189:2012).

[3] UNE-EN ISO/IEC 17025:2017 (2018). Requisitos generales para la competencia de los laboratorios de ensayo y calibración. (ISO/IEC 17025:2017).

[4] UNE-EN ISO 22870:2017 (2017). Análisis junto al paciente. Requisitos para la calidad y la competencia. (ISO 22870:2016).

